# Dissertation on Salivary Calculus

**Published:** 1844-09

**Authors:** William H. Dwinelle


### ARTICLE II.

*Dissertation on Salivary Calculus.* Read before the American Society of Dental Surgeons, at its Fifth Annual Meeting, held in the city of New York, July 17th, 1844. By WILLIAM H. DWINELLE.

THE subject of salivary calculus has been so variously treated upon by different authors, and in a manner so full and copious, that it would almost seem folly in me to bring it forward again, and especially, on an occasion like this, to make it the subject of a dissertation. But, I trust I shall be pardoned for the seeming presumption, when I introduce the subject to you in a new dress, and in a light not altogether usual, so that a part of it, at least, shall have the charm of novelty to atone for its various imperfections.

Salivary calculus is a calcareous incrustation, or deposit around the neck and sides of the teeth, found in the greatest abundance about those parts that are least subject to friction in mastication, or are contiguous to the salivary ducts. The manner of its deposit no longer admits of controversy; it is generally admitted, I believe, that it is deposited by the saliva, in which it is held in solution, as it flows from the parotid, submaxillary and sublingual glands, and the last glands, either from being more strongly impregnated with the substance, or yielding a greater flow of saliva, deposits a larger amount of the incrustation. It is mixed, more or less, with the mucus of the mouth, with which it incorporates itself. As it forms, it gradually hardens into a kind of cement, which varies in character according to the temperament, habits, health and other circumstances of the individual, each having a share in giving peculiarity to the formation. It assumes quite a variety of colors, white, yellow, brown, and not unfrequently almost black—indeed, we occasionally come across instances wherein nearly all these various shades are manifested in one individual, each particular shade formed in a layer by itself,—geologically speaking, making so many different strata in this *limestone formation!* It occasionally assumes a dark greenish color, though I am inclined to think, from numerous experiments, that this substance cannot be called calculus *proper*, having been able to discover but little or no lime in it; it may more properly be called dried or hardened mucus, mixed with the smallest quantity of earthy matter. The variousness of its texture and degrees of hardness is only equalled by its difference of color; some kinds of it being so hard as to require the strongest instruments, assisted by the direction of the most skilful hand, for its removal, while others are so soft and yielding as to be removed by, comparatively, the least effort. Its density invariably deepens with its color, the darker being more compact, while the lighter colored is more soft and porous. The teeth have no particular attraction for this deposit; any foreign substance retained in the mouth under similar circumstances, is just as liable to its attack; how often have we seen full, or parts of sets of artificial teeth, of those individuals who are not remarkable for cleanliness, completely incrustated with it. It is deposited so gradually that almost the least regard for cleanliness and decency will effectually insure one against its formation, and the injurious effects resulting from it. Some constitutions are more liable to it than others, yet every one is more or less subject to it; to us, this is comparatively inexplicable, though it is probably owing to some peculiarity of the system, which enables it to yield a more constant surplus of this earthy matter, for our observation will teach us, that those individuals who are remarkable for a sparseness of this quality in the system, such as rickety persons for instance, have little or no tartar on their teeth.

Various theories have been formed to explain the circumstance of the formation of salivary calculus. M. Delabarre assumed that it was a product of the gums and mucous membrane of the mouth while in an inflamed state, from the fact, that inflammation always accompanied the presence of tartar, when in quantities around the teeth, and also, from the fact, that it was found in greater quantities where the gums were inflamed the most. But this, it must be seen, is a very reversed mode of reasoning indeed, the inflammation of the gums is evidently occasioned by the accumulation of tartar itself, rather than that tartar is the *result* of inflammation. It would be like accounting for disease in the system by attributing it to the *pain* caused by disease! By the slightest observation, too, we will perceive that those parts of the teeth which are most subject to tartar, encounter the least friction in mastication; remove the tartar, have the patient observe habits of cleanliness, and the calculus will not be deposited, nor will the gums be inflamed.

It is a well known fact, I believe, that calculus is held in solution in most of the fluids of the body; it not unfrequently manifests itself in calcareous deposits in various parts of the system; in the stomach, liver, lungs, bladder, in the placenta of women, and even in the brain. Being suspended in the fluids of the body, in greater or less quantities, all that is necessary for its deposit is the presentation of some foreign substance under favorable circumstances, that may serve as a nucleus to which to attach itself; this may be some extraneous substance, presented immediately from without, or the result of some diseased action of the system.

The character of the food, and more particularly of the water, that is used by individuals, I am satisfied, has a very great influence in increasing or diminishing the quantity of the deposit of tartar on the teeth; observations made upon those who live in limestone districts, where the water that is used is highly charged with lime, and, upon those who live in lower regions, where the water is less impregnated with this earth, and approaches nearer the character of distilled water, will satisfy any one, that the fluids which are taken into the system have a material influence in regulating the quantity of the incrustation, and also in giving it character and quality. It is a generally admitted fact, that the character of the fluids used by individuals afflicted with calculi of the bladder, have a most evident influence upon them for better or worse, so much so that persons suffering under this disease have only found permanent relief by removing to another region, differing entirely in geological arrangement. Rickety children too, often manifest the most surprising change in their whole constitutional organization on being transferred from the low lands, to higher regions, where the water in its course is filtrated through bodies of limestone. What is it in the character of our mineral springs, which works such wonderful changes in children of this kind, if it is not in the large quantities of lime which they all, or nearly all, hold in solution. It is an admitted fact in materia medica, that to such children, lime in solution is given with the greatest success, it having a corrective effect in restoring a proper equilibrium between the lime and gelatine of the bone.

That saliva contains a greater amount of calcareous matter in solution, than the other fluids of the system, no one will doubt, when he analyzes the different fluids and compares the results with each other.

The immediate effect which tartar on the teeth has upon those organs, and upon the mouth, is, of course, familiar to you all. "Inflammation, turgescence and suppuration of the gums, inflammation of the alveolo-dental periostea, the destruction of the sockets and loss of the teeth, and an altered condition of the fluids of the mouth, are among the local effects that arise from the long continued presence of large collections of tartar on the teeth."* That there does exist the most intimate connexion and sympathy between the mouth and gums, and the various other parts of the system is no longer, I believe, a subject of discussion. It is unnecessary for me to do anything more than barely to refer to this subject, when it has been so ably and fully set forth in a paper, read before this society at our last annual meeting, by Professor Bond, of the Baltimore College of Dental Surgery.

After making repeated analysis of tartar, I have hardly succeeded in making any two agree; perhaps, however, as a general thing, the following may be relied upon as giving the usual proportions of the various substances which enter into its combination:

Of 100 parts, there are of

| | |
|---|---|
| Phosphate of lime, . . | 60 00 |
| Carbonate of lime, . . . . | 14 00 |
| Animal matter and mucus, . | 16 00 |
| Water and loss, . . . . | 10 00 |

The phosphate and carbonate of lime increase in quantity

* Harris on the Teeth, Gums, &c. Jour. Dental Science, vol. ii. p. 107.

with the depth of its color and density, and vice versa, the lighter colored containing less lime and more animal matter.

My experiments agree with M. Delabarre's, in regard to the action of acids upon this substance, "the dry black tartar, which is found in small quantities around the teeth of persons of good constitutions, dissolves with difficulty in muriatic acid;" indeed, as I have before remarked, in relation to this particular kind of tartar, it contains but little earthy matter, and approaches nearer in character, so far as regards earthy matter, to "that kind of soft white tartar, of mucous temperaments, scarcely at all soluble in acids, but which is readily dissolved in alkalies." The common yellow tartar is easily dissolved in most of the acids, even strong common vinegar dissolves it very readily; many practitioners seem to be aware of this fact, and use it freely in removing this substance from the teeth; but since it has been so conclusively proven by Dr. Westcott's admirable dissertation on this subject—published in the fourth volume of the Journal of Dental Science—that acids are mainly, if not entirely responsible for all dental caries, I think the propriety of this course, to say the least, is questionable.

I have been induced, by a notice in the December (1843) number of the Dental Journal, page 118, of a series of microscopic observations, made on the calculus of the teeth, by M. Mandl, of the French Academy of Science, to repeat the same, and see how far it was practicable to adopt his new theory, that "the tartar is nothing but a deposit of the skeletons of dead infusoria."

M. Mandl states that, "if we take some of the mucous matter which is accumulated upon and between the teeth, and dilute it with distilled water previously warmed, we shall immediately perceive a host of infusoria, which move about with great liveliness." I have tried this experiment repeatedly, but have as often failed in perceiving any indication of animal life, with the naked eye, but on bringing to my assistance a powerful microscope, the most demonstrable evidence of active life was manifested; numbers of infinitesimal beings were seen active with being, glorying in an existence, and performing all the sinuous evolutions and serpentine windings of bona fide snakes. I have ventured to give a sketch of two of my observations, and though they may not be minutely correct, will give a general idea of their character.*

Figure 1, represents an appearance, that with all my numerous experiments, I succeeded in obtaining but one; for this reason, I am not inclined to place too much confidence in this singular and unusual manifestation, or draw the inference that such animals do exist in the ordinary tartar of the teeth; true, I am unable, satisfactorily, to account for the presence of these animals here, but the fact that I could never produce a second appearance of them, forbids the conclusion that they belong to the organic structure of the tartar. That they may have been mixed with the food consumed by the individual, and afterwards become associated with the tartar, is not impossible; but, the fact, that they were mingled with the calculus, and *seemed* to be a part of its organization, is sufficient, I think, to warrant me in giving them, at least, a passing notice. The tartar, of which, this constituted a part, was of a yellowish brown color, was abundantly formed about the necks of the teeth, particularly the posterior parts of the inferior incisors, was taken from a person of bilious temperament, about forty-five years old. By reference to the drawing, you will perceive a number of egg-like substances, clustered together and attached to an irregular mass, which supports them; one of them is detached from the rest, presenting the same appearance as when I made the drawing. I found it, as well as the others in the cluster, to be semi-translucent in appearance, the light from the reflector of the instrument manifestly passing through it; it was of an oval form and somewhat corresponded with the *voloox* and the *cychdium* of Muller. Notwithstanding I made repeated observations with reference to the fact, I am not confident that I perceived any motion in the animalculæ here described, though it was perfectly evident that their position was changed from time to time, yet as I did not detect them in the *act*, I cannot vouch for their actual *moving*

* The sketch here alluded to, we are compelled to omit, for want of time to have a suitable engraving of it made. This we regret, as the drawing would add greatly to the interest of the dissertation, but as it did not come to hand until after the present number of the Journal had gone to press, we can do no better.—*Eds.*

existence. I found evidence enough, however, to convince me that they were real organized beings, yet their extreme minute size, even when powerfully magnified, prevented me from arriving at the most satisfactory conclusions in reference to their character, habits and general structure. I found, as represented in the drawing, something like the appearance of limbs attached to them, though hardly sufficiently regular as to warrant us in believing them such. It is probable they are nothing but small portions of fibrous matter that has become entangled with them, as there were other similar detached portions scattered over the field. In the calcareous mass, immediately under the cluster just referred to, there is a number of oval-shaped delineations, though somewhat faint and broken, corresponding with the appearance of the animalculæ above. If the evidence was not so imperfect in reference to this species of animalculæ, this would seem to justify us in adopting M. Mandl's theory, that "the earthy matter of the tartar of the teeth is composed entirely of the skeletons of dead infusoria."

There is another class of animalculæ, however, whose appearance forbids the least speculation as to their vitality or complete organization. They are represented in fig. 2, and are of the character I first referred to as being full of motion and active with life. They somewhat resemble vinegar eels in shape, but are shorter and more rigid in their motions; like the others, they are semi-translucent. This kind of animalculæ is easily obtained, as they abound in almost all kinds of tartar. I have enlarged the size of both views to better illustrate, particularly, that of fig. 1. In fig. 2, you will perceive I have given a more extensively magnified view of one of the animalculæ, in order that you may be better able to judge of their character and shape.

Strong vinegar, and the acids generally, unless very much diluted, is fatal to them. If a drop of the infusion of nut-galls, or tinct. of myrrh be placed upon them, they become rigid, and, after various spasms, assume a crescent shape, and, apparently, die; but they will soon become resuscitated, if an immediate application of a few drops of pure saliva be made to them—indeed, saliva seems to be the only element they will live in, as they die in any other liquid in a short time, even the purest rain water. They will exist in a temperature between 55 and 120, but will die in a very short time if their temperature be elevated or depressed above or below the two extremes. With the greatest care I have not exceeded in keeping them alive more than a few hours, and when once dead, I have discovered no means of resuscitating them, a peculiarity, however, that belongs to many other species of animalculæ, which, though perished and dry, even for years, immediately spring into life on coming in contact with fluids under favorable circumstances.

Animalculæ seem most abundant in calculus of a yellowish brown color, yet they abound in almost all kinds, presenting the same shape and character, with the exception of color, which, of course, invariably corresponds with the shade of the calculus with which they are associated. I have never been able to discover them in that "dark green tartar," which, from its acrid nature is so exceedingly destructible to the teeth.

My observations, assisted by a very excellent and powerful microscope, have not enabled me to discover the animalculæ as occupying more than a small part of the whole structure of the calculus, nor could I discover, from any indication, that the misshapen residue of earthy matter approached any thing in character to the "skeletons of dead infusoria." That a still further and more powerful magnification would develope animalculæ, or their skeletons, as occupying the whole structure and substance of calculus proper, I am not prepared to deny; indeed, reasoning from analogy, we would seem to be justified in this conclusion. Recent chemical and microscopic observations have placed the fact almost beyond a doubt, that most, if not all calcareous formations are composed of the shells of living and dead animalculæ or infusoria; chalk, many kinds of limestone, and other earthy formations have been found to be of this character; and I believe it an established and uncontroverted fact, that the coral rocks, which, while on the one hand they often spread the surface of the deep with wrecks of our proudest vessels, and on the other, rear themselves into beautiful islands to adorn its disk, and break in upon the monotony of the expanse with their delicate hues and fantastic shapes, are, but a conglomeration of skeletons of what were once living, moving beings.

These considerations might lead to a wide and beautiful field of investigation, and had I time, and were this the place for it, I would like to enter upon that field; excuse me while I digress a moment barely to hint at it, and I will close.

When I consider the abundant and constantly increasing evidence we have on the subject, it requires but a little stretch of the imagination to adopt a theory; that every particle of matter, however minute, is endowed with a latent principle of life, that only waits favorable circumstances to burst forth into actual existence; a principle of life imparted to it at the creation of matter itself, and which it can never lose, however often it may assume change or modification. That organized forms may undergo decomposition, and even become imperceptibly divided, but the alternate particles of which they are composed never lose their vitality. That every particle of matter has an instinctive tendency to break the bonds of its association with organized matter, and assume an independent and moving existence. The air we breathe, the water we drink, and the food we take into our system, is, in its ultimate particles, completely impregnated with the principle of life; true, they may not as yet, either by combination or separation, have assumed a quickened and independent existence, but they only await the proper influence of circumstances, to spring into actual life, and assume their place among created beings. One of the principal of these resuscitating or vitalizing influences is *heat*, under proper modification and circumstances.

Animal matter, such as meat, under the favorable influence of heat, properly directed, notwithstanding it may not have the least possible opportunity of communicating with any external substance, will, in its decomposition, after a given time, be transformed into numberless living beings. Vegetable matter, bread for instance, under the same circumstances, will produce like results. Many of the fluids of the bodies of all animals contain living animalculæ, perhaps none, however, more plentifully than in the generative fluid, in which it abounds in the greatest abundance; here we find them striking at the very root of life, anticipating new forms of being, they have commenced their attack and work of destruction at this early period. We see, too, from the result of a series of experiments, an account of which I have just laid before you, that salivary calculus, is composed, at least, in part, of living, moving beings; and from the success that has crowned my efforts in this very partial and imperfect investigation, I am not prepared to deny that a more complete and wisely directed course of experiments, by an abler hand, will place the matter beyond controversy, that, as declared by M. Mandl, "the tartar (proper) of the teeth is composed entirely of living and dead animalculæ."

Viewing things in this light, we behold myriads of infinitesimal beings surrounding and besetting us on every hand, within and without, warring against our existence, and against which, it is the effort of nature to struggle; breaking in and infringing upon the organs of vitality, until they become so interrupted and crippled in their efforts, that sinking nature can no longer resist, and total decomposition follows as a matter of course. Then, when life, the great preservative of the body, that exercises control over matter, and holds it together in harmonious connexion, when this power of resistance shall be overcome by this warring, living principle, within and without, then will the harmony be disturbed, the great chain of affinity that binds parts together in one great whole, will be broken; affinities shall cease; the law of attraction shall be at an end, and the principle of repulsion shall rule in its place; the body dies, and corruption has its sway. But here, upon all this ruin, another race of beings must have their brief existence, go through the routine of their short hour and die, and be resolved into new forms of being with the rest; an existence purchased by the dormancy of years, enveloped in the womb of matter; an existence the result of their victory over life, still an existence though bought at the price of their more certain death. Affinities, too, with them shall cease, and the ultimate, yet living particles of matter will be left free to the embracing influence of attraction to be resolved into new forms of being.

But I am reminded, that, although, I have but briefly alluded to the subject, I have, perhaps, already digressed too far to render this dissertation of practical utility, and I will close with the hope that the mere hint I have here given will be suggestive of more enlarged views and conceptions to those who may hereafter pursue this beautiful field of investigation. To those of us who are fond of the curious, and whose hearts thrill with pleasure at the displays of the wisdom and excellence of God, manifested in all-glorious nature, will find this subject giving out its copious reward at every step of our advancement.

